# A Comparison on Prevalence of Hypertension and Related Risk Factors between Island and Rural Residents of Dalian City, China

**DOI:** 10.1155/2019/6413102

**Published:** 2019-05-13

**Authors:** Baiting Liu, Hainiang Liu, Rongmei Na, Xiaofei Li, Qianxiao Li, Libo Chen, Wencheng Tu, Jiahui Hu, Dong Cheng, Yalan Cao, Zhu Li, Weiyi Fang, Ning Zhu, Qin Yu

**Affiliations:** ^1^Department of Cardiology, Affiliated Zhongshan Hospital of Dalian University, Dalian 116001, China; ^2^Medical College, Dalian University, Dalian 116022, China; ^3^Linqu County Procuratorate, Weifang 262600, China; ^4^Department of Cardiology, Zhejiang Province Hospital of Integrated Traditional Chinese and Western Medicine, Hangzhou 310012, China; ^5^Department of Cardiology, People's Hospital of Jilin City, Jilin 132000, China; ^6^Department of Cardiology, Jingmen No.1 People Hospital, Jingmen 448000, China; ^7^Department of Cardiology, Suihua No.1 Hospital, Suihua 152000, China; ^8^Medical College, Zunyi University, Zunyi 563100, China; ^9^Department of Cardiology, Zhuanghe Central Hospital, Dalian 116400, China; ^10^Department of Cardiology, Shanghai Chest Hospital, Shanghai 200030, China; ^11^Department of Cardiology, The Second Affiliated Hospital of Dalian Medical University, Dalian 116023, China

## Abstract

This study aimed to compare the prevalence of hypertension between the island and rural residents in Dalian, China, and to explore associated risk factors of hypertension in order to provide evidence for the establishment of targeted strategy of hypertension prevention and treatment for island and rural residents. The modified MONICA questionnaire survey was performed on 7764 island and rural residents aged ≥40 years (including 2652 island residents and 5112 rural residents). Our data showed that totally weighted prevalence of hypertension was significantly higher in rural residents than in island residents (61.9% vs. 55.2%, P<0.001). Multivariate binary logistic regression analysis showed that older age, higher BMI, lower education level, and higher LDL-C and UA levels were independently associated with increased risk of having hypertension both in island and in rural residents. The weighted awareness rate (29.9% vs. 17.3%, P<0.001), treatment rate (51.4% vs. 28.5%, P<0.001), and control rate (36.3% vs. 24.0%, P=0.001) of hypertension were all significantly higher in island residents than those in rural residents. In conclusion, our survey shows that the epidemics of hypertension are extremely high in surveyed residents in island and rural areas of Dalian city, while awareness, treatment, and control rats of hypertension in these residents are much lower than the national level. The scenario is even worse in rural residents as compared with island residents of Dalian, China.

## 1. Introduction

The prevalence of hypertension is increasing constantly in mainland China in line with the population aging process and rapid economic development over recent decades. Hypertension is remarkably related to increased risk of cardiovascular comorbidities and mortality [1]. Based on a national survey of the prevalence of hypertension in China between 2012 and 2015, 23.2% of the Chinese adult population ≥18 years of age suffered from hypertension [2]. There is a significant difference in prevalence of hypertension in various regions of China due to the impact of complex geographical patterns and economic and cultural diversity. For example, in economically developed regions, the prevalence of hypertension is significantly higher among rural residents than among urban residents (31.3% vs. 29.2%, p* *=* *0.001), whereas this disparity in the prevalence of hypertension between urban and rural areas disappeared in the northern region (31.6% vs. 31.2%, p* *=* *0.505) [3]. Also, according to the recent data, similar prevalence of hypertension between urban and rural residents in China was reported (23.4% vs. 23.1%, P=0.819) [2]. Therefore, it is of importance to investigate region-related prevalence and related risk factors of hypertension in various regions in order to help formulate and devise local public health strategies and approaches in the prevention and management of hypertension.

In 2012, we conducted an epidemiological survey on prevalence of hypertension on permanent residents living in an island encircled by the Yellow Sea (Zhangzi Island) and residents living on a coast district near the Yellow Sea (Zhuanghe District). Zhangzi Island is one of the islands in the Yellow Sea and 55 km from Zhuanghe District. Most residents on Zhangzi Island live on fishing and fisheries processing. Zhuanghe District lies in the north coast of the Yellow Sea. Most residents in Zhuanghe District live on farming. In present study, we investigated the epidemic features and risk factors of hypertension in the island (Zhangzi Island) and rural (Zhuanghe District) residents; our data might be helpful in the establishment of more effective and targeted prevention and management strategies for residents living in these areas.

## 2. Methods

### 2.1. Study Population

A total of 8347 permanent residents aged ≥18 years in Zhangzi Island and Zhuanghe District took part in this survey. Proportion of participants aged <40 years was 10.8% in the island area and 4.7% in the rural area, respectively. Most of young male residents both in island and in rural areas were absent at the time of survey because they lived outside of their hometown for work. Thus, the majority of participants comprised of female and middle-aged or elderly male residents. Eventually, 2652 island residents (716 male and 1936 female) and 5112 rural residents (1750 male and 3362 female) aged ≥40 years were included in this study for the final analysis.

### 2.2. Questionnaire Survey

“Survey Questionnaire of Cardiovascular Disease Risk Factors” derived from amendatory MONICA study was used in this study [4, 5]. The survey staffs received training on data collection before the survey.

### 2.3. Definitions

Blood pressure was measured as previously described [6]. Briefly, blood pressure was measured twice by trained examiners following a standardized protocol using aneroid sphygmomanometers. Participants sat with both feet on the floor for at least five minutes before the first measurement. The two blood pressure measurements were taken at least two minutes apart. Hypertension is defined according to “The Seventh Report of the Joint National Committee on Prevention, Detection, Evaluation, and Treatment of High Blood Pressure” (JNC 7) [7]. Patients were divided into 2 subgroups as follows: Group 1, normotensive participants (i.e., no medical history of hypertension and SBP <140 mmHg and DBP <90 mmHg measured at the survey time); Group 2, hypertensive participants (self-reported hypertension with or without antihypertensive medications use or SBP≥140 mmHg and/or DBP≥90 mmHg measured at the survey time). Weight status was defined by body mass index (BMI) according to the lower cutoff values recommended by WHO experts for Asians, i.e., overweight as BMI ≥ 24 kg/m^2^ and obesity as BMI ≥ 28 kg/m^2^ [8–10]. Participants who were currently smoking cigarettes, bides, or hookah with an average of more than 1 cigarette daily were defined as current daily smokers [11]. Participants who were consuming alcohol within the past 1 year and daily alcohol consumption (alcohol content) ≥25g in men and ≥15g in women were defined as current alcohol drinkers [12]. The highest education level of participants was categorized into 5 levels: illiterate or semiliterate, primary education, secondary education, upper secondary education, and tertiary or higher education.

### 2.4. Biochemistry Examination

Blood samples were taken from all participants at the time of survey. Laboratory analysis including serum total cholesterol (TC), triglyceride (TG), low-density lipoprotein cholesterol (LDL-C), high-density lipoprotein cholesterol (HDL-C), urea, creatinine, and uric acid (UA) was performed. Hyperuricemia was defined if serum UA >420 *μ*mol/L in men and postmenopausal women and serum UA >360 *μ*mol/L in premenopausal women according to the current recommendations of Chinese experts consensus [13, 14].

### 2.5. Statistical Analysis

Continuous variables were expressed as mean ± standard deviation (SD) or median (quartiles). Differences on continuous variables between groups were compared using unpaired two-sample Student's* t*-test after normalization if indicated. Nonnormally distributed variables were compared using Mann-Whitney U test. Categorical variables were compared across groups using a Chi-square test for the overall test and column proportions were compared using z-test. Multivariate binary logistic regression analysis was conducted to determine independent risk factors of hypertension in this cohort. Adjusted odds ratios (ORs) with 95% confidence interval (CI) were calculated. Survey data were weighed based on the Sixth National Population Census of the People's Republic of China in 2010 [15] to calculate weighted prevalence, awareness rate, treatment rate, and control rate of hypertension. A significance level of 0.05 was used. Statistical analysis was performed using IBM SPSS, version 22 for Windows (SPSS).

### 2.6. Ethical Consideration

Ethical approval was obtained from the Institute Ethical Committee of Zhongshan Hospital of Dalian University. All the participants signed informed consent.

## 3. Results

### 3.1. Demographic Data

Table 1 shows the age and sex distribution data of island and rural residents.

### 3.2. Weighted Prevalence of Hypertension Stratified by Age and Sex

The systolic blood pressures (SBP) and diastolic blood pressures (DBP) in island and rural residents are shown in Table 2. SBP (144 ± 18 vs. 144 ± 18 mmHg, P>0.05) and DBP (92 ± 9 vs. 92 ± 9 mmHg, P>0.05) were similar between island and rural residents with hypertension.

Totally weighted prevalence of hypertension was significantly higher in rural residents than in island residents (61.9% vs. 55.2%, P<0.001). As shown in Table 3, weighted prevalence of hypertension in island residents was 55.2% (95% CI 53.3-57.0%) and was significantly higher in males than in females (61.2% vs. 48.8%, P<0.001). The prevalence of hypertension in rural residents was 61.9% (95% CI 60.5-63.2%) and was similar between males and females (61.3% vs. 62.6%, P=0.561).

As expected, the prevalence of hypertension increased with age in both island and rural groups. The hypertension prevalence was significantly lower in island residents than in rural residents at 50-59, 60-69, and 70-79 year age groups, respectively. Hypertension prevalence remained unchanged in various groups of age in male residents, while hypertension prevalence increased continuously after age 50 in female residents living both in island area and in rural area.

### 3.3. Blood Parameters

As shown in Table 4, serum TC, TG, LDL-C, urea, creatinine, and UA levels were significantly higher and HDL-C was significantly lower in island residents than in rural residents. Serum TC, TG, LDL-C, urea, creatinine, and UA levels were significantly higher in the hypertensive group than in the normotensive group both in island residents and in rural residents. HDL-C was significantly lower in the hypertensive group than in the normotensive group in island residents, while it was similar between groups in rural residents.

### 3.4. BMI

BMI of island residents was higher in island residents than in rural residents (25.1 ± 3.5 vs. 24.3 ± 3.41, P<0.001). The prevalence of hypertension increased with increasing BMI both in island residents (33.3% in underweight, 44.6% in normal BMI, 55.7% in overweight, and 74.6% in obesity, respectively) and in rural residents (45.7%, 54.2%, 66.4%, and 80.1%, respectively, Table 5).

### 3.5. Smoking and Alcohol Drinking

In surveyed island residents, proportions of smoking and alcohol drinking were 13.5% (358/2652) and 12.8% (340/2652), respectively. In surveyed rural residents, proportions of smoking and alcohol drinking were 19.3% (998/5112) and 12.7% (651/5112).

The weighted prevalence of hypertension was similar between no smoking group and smoking group both in island residents (no smoking 55.1% vs. smoking 55.4%, P=0.963) and in rural residents (62.9% vs. 59.4%, P=0.071).

The weighted prevalence of hypertension was significantly higher in alcohol drinking group than in no alcohol drinking group in island residents (alcohol drinking 64.7% vs. no alcohol drinking 52.9%, P<0.001), while it remained similar between groups in rural residents (61.4% vs. 63.5%, P=0.187).

### 3.6. Education Levels

Proportions of illiterate or semiliterate, primary education, lower secondary education, upper secondary education, and tertiary or higher education were 11.7%, 37.8%, 41.9%, 5.7%, and 2.9% in island residents and were 20.3%, 39.2%, 31.2%, 8.1%, and 1.3% in rural residents, respectively.

The prevalence of hypertension decreased with increase in education levels both in island residents (68.5% vs. 64.7% vs. 46.1% vs. 42.4% vs. 39.5%, P<0.001) and in rural residents (70.3% vs. 57.4% vs. 54.1% vs. 57.9% vs. 44.7%, P<0.001). Among residents received upper secondary or higher education, the prevalence of hypertension was significantly lower in the island group than in the rural group (41.4% vs. 56.1%, P=0.001), while it was similar between island and rural residents who received lower secondary or lower education (56.7% vs. 59.1%, P=0.062).

### 3.7. Awareness Rate, Treatment Rate, and Control Rate of Hypertension

As shown in Table 6, the weighted awareness rate (29.9% vs. 17.3%, P<0.001), treatment rate (51.4% vs. 28.5%, P<0.001), and control rate (36.3% vs. 24.0%, P=0.001) of hypertension were all significantly higher in island residents than those in rural residents.

As shown in Table 7, calcium channel blockers were most frequently used both in island and in rural residents (42.1% vs. 22.8%, P<0.001). The survey results showed that most hypertensive patients took mono antihypertensive agent both in island and in rural areas. The proportion of combined antihypertensive medication is significantly higher in island residents than in rural residents (32.4% vs. 19.8%, P<0.001). In addition, 47.1% of island residents and 57.1% of rural residents took other nonstandard medications, mostly the Chinese herb medicine.

### 3.8. Independent Risk Factors of Hypertension in Island and Rural Residents

Multivariate binary logistic regression analysis showed that older age, higher BMI, lower education level, and higher LDL-C and UA levels were independently associated with increased risk of having hypertension both in island and in rural residents (Tables 8 and 9). Female sex remained as independent risk factor of hypertension in island residents.

## 4. Discussion

The major findings of this study included that (1) the prevalence of hypertension adopting JNC 7 guideline was 61.9% in residents of Zhuanghe District (rural area) and it was significantly higher than in residents of Zhangzi Island (island area, 55.2%, P<0.001); (2) older age, higher BMI, lower education level, and higher LDL-C and UA levels were independently associated with increased risk of having hypertension both in island and in rural residents.

### 4.1. Prevalence and Independent Risk Factors of Hypertension in Surveyed Areas

According to a nationwide survey data from 2012 to 2015, weighted prevalence of hypertension in Chinese adult population aged ≥18 years was 23.2% [2]. Total prevalence of hypertension in Liaoning Province was 28.6% and 30.8% in urban residents and 26.2% in rural residents, respectively [2]. Our survey data showed that the weighted prevalence of hypertension in the island residents aged ≥ 40 years was 61.9% and 55.2% in the rural residents. This prevalence was also higher than nationwide prevalence in community-dwelling adults aged 35-75 years (44.7%) [15]. The awareness rate, treatment rate, and control rate of blood pressure were 46.5%, 41.1%, and 13.8%, respectively, in China. Our survey results showed that the awareness rate, treatment rate, and control rate of hypertension in two surveyed areas are significantly lower than national level. The weighted awareness rate in island residents was 29.9%, and 51.4% of them were receiving antihypertensive medications, and among treated patients, control rate was 36.3%. The awareness rate, treatment rate, and control rate in rural residents were 17.3%, 28.5%, and 24.0%, respectively, and were significantly lower than those in island residents. The following points might relate to the alarming hypertension epidemics both in island and in rural areas reported in this study.

The educational level might be responsible for the high prevalence of hypertension. Educational level in the two areas is under average national level; 91.4% population in island and 93.2% population in rural area are mainly junior middle school level or below [16]. Previous survey found that awareness, treatment, and control rates of hypertension were higher in urban residents compared with rural residents, and low education level was associated with lower rates of awareness, treatment, and control rats of hypertension. The slightly better education level in island residents might, therefore, be responsible for slightly better scenario on the higher awareness, medicine adherence, and control rates of hypertension in island residents as compared to the residents in rural area.

Aging is related to higher prevalence in these two surveyed areas, which is in line with the domestic related conclusions [17]. Besides above factors, higher BMI and higher LDL-C and UA levels are found to be the independent risk factors of hypertension in residents of the surveyed residents, in line with previous reports [18–22].

### 4.2. Treatment and Medication Status

The most common antihypertensive medications included CCB (nifedipine), beta-blocker, and ACEI in island residents and CCB, diuretic, and ACEI in rural residents. It is difficult for most patients to take mono antihypertensive drug to control the hypertension and reach individualized treatment. It is incompatible with the advocated principle of combining of antihypertensive drugs. The antihypertensive effect is not ideal; the island control rate of hypertension is only 36.3%, even 24.0% in the rural area. The slightly better control rate in island hypertensive residents might relate to the factor that the proportion of combined antihypertensive medication was significantly higher in island residents than in rural residents (32.4% vs. 19.8%, P<0.001). While the general unacceptable low hypertension control rate might be related to the widespread use of nonstandard medications, especially the Chinese herb medicine in surveyed hypertensive residents, we found that 47.1% of island residents and 57.1% of rural residents took nonstandard medications to treat their hypertension, mostly the Chinese herb medicine.

## 5. Conclusion

In conclusion, our survey shows the epidemics of hypertension are extremely high in surveyed residents both in island and in rural areas of Dalian city, while awareness, treatment, and control rates of hypertension in these residents are much lower than the national level. Targeted strategies including health education and standardized hypertension treatment are warranted to reduce the hypertension burden in these areas.

## Figures and Tables

**Table 1:** The age and sex distribution in residents living in the island and rural areas.

Age (years)	Island			Rural		
	Male	Female	Total	Male	Female	Total
	N (%)	N (%)	N (%)	N (%)	N (%)	N (%)
40-49	72 (10.1)	513 (26.5)	585 (22.1)	165 (9.4)	720 (21.4)	885 (17.3)
50-59	128 (17.9)	659 (34.0)	787 (29.7)	368 (21.0)	1051 (31.3)	1419 (27.8)
60-69	304 (42.5)	521 (26.9)	825 (31.1)	707 (39.9)	1065 (60.1)	1772 (34.7)
70-79	166 (23.2)	199 (10.3)	365 (13.8)	394 (22.5)	457 (13.6)	851 (16.6)
≥80	46 (6.4)	44 (2.3)	90 (3.4)	116 (6.6)	69 (2.1)	185 (3.6)

Sum	716	1936	2652	1750	3362	5112

**Table 2:** The systolic and diastolic blood pressures in island and rural residents (mmHg).

	Island			Rural		
	Normotensive	Hypertensive	Total	Normotensive	Hypertensive	Total
SBP	116 ± 10	144 ± 18*∗*	132 ± 21	116 ± 11	144 ± 18*∗*	133 ± 21
DBP	75 ± 7	92 ± 9*∗*	84 ± 12	76 ± 6	92 ± 9*∗*	86 ± 11†

*∗* P<0.05 vs. normotensive; † P<0.05 vs. Island. SBP: systolic blood pressure; DBP: diastolic blood pressure.

**Table 3:** Weighted prevalence of hypertension stratified by age and sex [% (95% CI)].

		Island			Rural	
	Males	Females	Total	Males	Females	Total
Age (years)						
40-49	59.7 (47.5-71.7)	34.1*∗* (30.0-38.4)	47.7 (44.7-50.7)	52.7 (44.8-60.5)	47.6*∗* (43.9-51.4)	50.6 (48.2-53.0)
50-59	61.7 (52.7-70.2)	53.6*∗* (49.7-57.4)	56.6 (53.5-59.6)	63.0 (57.9-68.0)	64.4 (61.4-67.3)	63.9† (61.6-66.1)
60-69	61.2 (55.5-66.7)	62.8 (58.5-66.9)	61.2 (55.5-66.7)	63.9 (60.3-67.5)	71.4*∗* (68.5-74.1)	63.9† (60.3-67.5)
70-79	60.8 (53.0-68.3)	65.8 (58.8-72.4)	63.6 (58.4-68.5)	72.1 (67.4-76.5)	77.7 (73.6-81.4)	75.1† (72.0-78.0)
80-99	78.3 (63.6-89.1)	72.7 (57.2-85.0)	75.6 (65.4-84.0)	69.8 (60.6-78.0)	76.8 (65.1-86.1)	73.6 (67.7-78.9)
Total	61.2 (58.7-63.7)	48.8*∗* (46.2-51.5)	55.2 (53.3-57.0)	61.3 (59.5-63.0)	62.6 (60.6-64.5)	61.9† (60.5-63.2)

*∗* P<0.05 vs. Males; † P<0.05 vs. Island.

**Table 4:** The blood biochemical parameters in island and rural residents.

	Island			Rural		
	Normotensive	Hypertensive	Total	Normotensive	Hypertensive	Total
TC (mmol/L)	5.25 ± 1.01	5.45 ± 1.07*∗*	5.36 ± 1.05	5.04 ± 0.89	5.15 ± 0.91*∗*	5.11 ± 0.90†
TG (mmol/L)	1.24 ± 0.71	1.42 ± 0.85*∗*	1.34 ± 0.80	1.15 ± 0.72	1.29 ± 0.87*∗*	1.23 ± 0.82†
HDL-C (mmol/L)	1.36 ± 0.32	1.33 ± 0.33*∗*	1.34 ± 0.32	1.38 ± 0.43	1.37 ± 0.38	1.37 ± 0.40†
LDL-C (mmol/L)	2.40 ± 0.61	2.56 ± 0.70*∗*	2.49 ± 0.67	2.23 ± 0.57	2.30 ± 0.63*∗*	2.27 ± 0.61†
Urea (mmol/L)	6.26 ± 1.53	6.57 ± 2.13*∗*	6.43 ± 1.90	6.04 ± 1.60	6.25 ± 1.78*∗*	6.17 ± 1.71†
CREA (*μ*mol/L)	66.6 ± 16.1	72.2 ± 52.9*∗*	69.7 ± 40.9	64.8 ± 12.7	65.9 ± 16.4*∗*	65.4 ± 15.0†
UA (*μ*mol/L)	317 ± 90	351 ± 92*∗*	336 ± 92	291 ± 70†	303 ± 75*∗*	298 ± 73†

*∗* P<0.05 vs. Normotensive; †P<0.05 vs. Island. TC: total cholesterol; TG: triglyceride; HDL-C: high-density lipoprotein cholesterol; LDL-C: low-density lipoprotein cholesterol; CREA: creatinine; UA: uric acid.

**Table 5:** Weighted prevalence of hypertension stratified by BMI in island and rural residents.

		Island		Rural	P value
BMI (kg/m^2^)	Mean ± SD	25.1±3.5	Mean ± SD	24.3 ± 3.4	<0.001
	No.	Prevalence of HP	No.	Prevalence of HP	<0.001
Underweight	13/43	33.3 (20.0-49.0)	60/145	45.7 (37.8-53.7)	
Normal	464/1032	44.6 (41.7-47.6)	1205/2328	54.2 (52.2-56.2)*∗*	
Overweight	556/994	55.7 (52.7-58.7)†‡	1245/1908	66.4 (64.2-68.5) *∗*†‡	
Obesity	434/581	74.6 (71.0-77.9)†‡§	576/727	80.1 (77.0-83.0) †‡§	
P value		<0.001		<0.001	

*∗* P<0.05 vs. Island; † P<0.05 vs. underweight; ‡ P<0.05 vs. Normal; § P<0.05 vs. Overweight. BMI: body mass index; HP: hypertension.

**Table 6:** Awareness rate, treatment rate, and control rate of hypertension in island and rural residents.

	No.	Island	No.	Rural	P value
		(%, 95% CI)		(%, 95% CI)	
Awareness rate	805/2649	29.9 (28.2-31.6)	848/5107	17.3 (16.3-18.3)	<0.001
Treatment rate	414/2649	15.4 (14.1-16.7)	246/5107	4.9 (4.4-5.6)	<0.001
Within awareness group	414/805	51.4 (48.0-54.8)	246/848	28.5 (25.6-31.6)	<0.001
Control rate	147/2649	5.6 (4.8-6.5)	57/5107	1.2 (0.9-1.5)	<0.001
Within treatment group	147/414	36.3 (31.8-40.9)	57/247	24.0 (19.0-29.7)	0.001

**Table 7:** Hypertensive mono medication status in island and rural residents.

	Island	Rural	P value
Diuretic	8.7% (36/413)	19.1% (47/246)	<0.001
Beta-blocker	20.5% (85/414)	10.1% (25/247)	<0.001
CCB	42.1% (174/413)	22.8% (56/246)	<0.001
ACEi	14.8% (61/413)	17.9% (44/246)	0.290
ARB	4.3% (18/414)	1.6% (4/246)	0.060
Others	47.1% (195/414)	57.1% (141/247)	0.013

CCB: calcium channel blockers; ACEi: angiotensin converting enzyme inhibitors; ARB: angiotensin II receptor blockers.

**Table 8:** Multivariate binary logistic regression analysis of risk factors of hypertension in island residents.

	Wald	P value	OR	95% CI for OR	
				Lower	Upper
Age (years)					
40-49	37.480	<0.001	Reference	-	-
50-59	7.850	0.005	1.333	1.090	1.630
60-69	15.434	<0.001	1.609	1.269	2.040
70-79	12.024	0.001	1.687	1.255	2.268
≥80	22.161	<0.001	3.302	2.008	5.429
Male vs. female	9.077	0.003	1.353	1.111	1.647
BMI					
Underweight/normal	99.762	<0.001	Reference	-	-
Overweight	19.956	<0.001	1.517	1.263	1.821
Obesity	99.388	<0.001	3.299	2.609	4.172
Alcohol drinking	3.302	0.069	1.235	0.984	1.551
Education levels					
Lower secondary or lower vs. upper secondary or higher education	15.667	<0.001	1.805	1.347	2.419
TG (mmol/L)	2.993	0.084	1.104	0.987	1.234
LDL-C (mmol/L)	16.206	<0.001	1.295	1.142	1.469
Urea (mmol/L)	0.406	0.524	1.015	0.969	1.064
UA (umol/L)	25.202	0.001	1.003	1.002	1.004
Constant	103.128	0.001	0.052		

OR: odds ratio; CI: confidence interval; BMI: body mass index; TG: triglyceride; LDL-C: low-density lipoprotein cholesterol; UA: uric acid.

**Table 9:** Multivariate binary logistic regression analysis of risk factors of hypertension in rural residents.

	Wald	P value	OR	95% CI for OR	
				Lower	Upper
Age (years)					
40-49	138.232	<0.001	Reference	-	-
50-59	50.701	<0.001	1.821	1.544	2.147
60-69	56.344	<0.001	2.186	1.782	2.681
70-79	77.681	<0.001	3.521	2.662	4.659
≥80	29.730	<0.001	3.437	2.205	5.356
Male vs. female	0.084	0.772	1.026	0.864	1.217
BMI					
Underweight/normal	126.283	<0.001	Reference	-	-
Overweight	67.953	<0.001	1.894	1.627	2.204
Obesity	94.646	<0.001	3.346	2.623	4.268
Alcohol drinking	0.509	0.476	1.075	0.881	1.313
Education levels					
Lower secondary or lower vs. upper secondary or higher education	0.190	0.663	1.055	0.829	1.343
TG (mmol/L)	13.057	<0.001	1.190	1.083	1.308
LDL-C (mmol/L)	7.730	0.005	1.188	1.052	1.340
Urea (mmol/L)	7.042	0.008	1.062	1.016	1.109
UA (umol/L)	0.617	0.432	1.000	0.999	1.001
Constant	38.604	<0.001	0.200		

OR: odds ratio; CI: confidence interval; BMI: body mass index; TG: triglyceride; LDL-C: low-density lipoprotein cholesterol; UA; uric acid.

## Data Availability

The data used to support the findings of this study are available from the corresponding author upon request.
